# The transcription factor CsbHLH18 of sweet orange functions in modulation of cold tolerance and homeostasis of reactive oxygen species by regulating the antioxidant gene

**DOI:** 10.1093/jxb/ery065

**Published:** 2018-02-21

**Authors:** Jingjing Geng, Ji-Hong Liu

**Affiliations:** Key Laboratory of Horticultural Plant Biology (MOE), College of Horticulture and Forestry Sciences, Huazhong Agricultural University, Wuhan, China

**Keywords:** Basic helix–loop–helix transcription factors, *Citrus sinensis*, cold stress tolerance, E-box element, genome-wide analysis, ROS homeostasis

## Abstract

The basic helix–loop–helix (bHLH) transcription factors (TFs) comprise one of the largest gene families in plants, and participate in various physiological processes, but the physiological role and regulatory function of the majority of bHLHs remain poorly understood. Here, a total of 56 putative *CsbHLH* genes were identified in sweet orange (*Citrus sinensis*) based on a genome-wide analysis. The *CsbHLH* genes, except four members, were distributed throughout nine chromosomes and divided into 19 subgroups. Most of the *CsbHLH* genes were responsive to cold stress, with the greatest up-regulation being observed in *CsbHLH18*. CsbHLH18 is localized in the nuclei and has transcriptional activation activity. Overexpression of *CsbHLH18* conferred enhanced cold tolerance in transgenic tobacco. The transgenic plants accumulated significantly less reactive oxygen species (ROS), concurrent with increased activities and transcript levels of antioxidant enzymes. In contrast, knockdown of *bHLH18* by RNAi in trifoliate orange promoted cold susceptibility, accompanied by down-regulation of antioxidant genes and accumulation of more ROS. Protein–DNA interaction assays demonstrate that CsbHLH18 directly and specifically binds to and activates the promoter of *CsPOD*. Taken together, these findings indicate that *CsbHLH18* plays a positive role in cold tolerance through, at least partly, modulation of ROS homeostasis by directly regulating the antioxidant gene.

## Teaser

CsbHLH18, a bHLH transcription factor of *Citrus sinensis,* functions as a positive regulator of cold tolerance due to promoted ROS scavenging, and directly targets a *POD* gene.

## Introduction

Plants are routinely exposed to a range of abiotic factors, such as extreme temperatures, drought, and high salinity, which have adverse effects on plant growth and may substantially reduce crop productivity. Plants have also evolved a range of complex mechanisms to tolerate the harsh environmental stresses (Huang *et al.*, 2013; Zhu, 2016; Zandalinas *et al.*, 2018). Stress responses are known to involve numerous signaling pathways that can form intricate networks composed of various genes encoding structural and regulatory proteins that play direct or indirect roles in protecting plants against the abiotic stresses (Lata and Prasad, 2011; Liu *et al.*, 2014; Li *et al.*, 2017; Zhao *et al.*, 2017). Transcription factors (TFs) are important regulatory proteins that function to control the expression of target genes through binding to specific *cis*-acting elements within the promoters (Golldack *et al.*, 2011). Therefore, identification and characterization of stress-responsive TFs is crucial to elucidate the molecular network associated with stress response and has been also widely adopted as a strategy for unraveling valuable candidate genes for improving stress tolerance using genetic engineering (Liu *et al.*, 2014; Zhu, 2016).

The basic helix–loop–helix (bHLH) proteins are one of the largest TF families (Riechmann *et al.*, 2000; Carretero-Paulet *et al.*, 2010). They typically consist of 50–60 amino acids that form two functionally distinct parts, the basic region at the N-terminus and the HLH region at the C-terminus. The basic region contains ~15 amino acids with DNA binding ability, which allows the bHLH proteins to bind to the consensus hexa-nucleotide (5'-CANNTG-3') called the E-box element (Atchley *et al.*, 1999). The HLH region is composed of two amphipathic helices connected by a divergent loop, and functions in facilitating formation of homo- and/or heterodimeric complexes between proteins (Nair and Burley, 2000; Buck and Atchley, 2003).

The *bHLH* genes act as regulatory components in transcriptional networks, and are involved in many diverse biological processes, such as fruit dehiscence and flowering (Ito *et al.*, 2012), hormone signaling (Friedrichsen *et al.*, 2002), and trichome and root development (Menand *et al.*, 2007). In addition, an increasing number of studies demonstrate that the bHLHs also function in responses to a range of stresses. For example, *Arabidopsis thaliana* INDUCER OF CBF EXPRESSION1 (ICE1) and ICE2 and their homologs in several different plants are reported to play key roles in mediating cold responses (Chinnusamy *et al.*, 2003; Feng *et al.*, 2012; Huang *et al.*, 2013). RERJ1 and *OsbHLH148* of rice are proposed to be associated with wounding and drought responses (Kiribuchi *et al.*, 2005; Seo *et al.*, 2011). In addition, a few bHLH proteins are reported to be involved in modulation of nutrient homeostasis (Sivitz *et al.*, 2012; Zhao *et al.*, 2016). However, it is worth mentioning that although tremendous progress has been achieved concerning the functional characterization of *bHLH* genes in a diversity of plant species, the physiological role and regulatory function of many bHLH TFs, particularly those of non-model woody fruit trees, are still poorly understood.

Sweet orange (*Citrus sinensis*) accounts for more than half of the total citrus production worldwide. However, cold sensitivity is a major factor limiting sustainable development of the sweet orange industry, and there is considerable interest in fortifying the cold tolerance in this species. Due to unique reproductive barriers caused by polyembryony, sweet orange germplasm enhancement via traditional cross-breeding is difficult, and genetic engineering offers an alternative approach in the breeding pipeline. The published genome of sweet orange (Xu *et al.*, 2013) provides a valuable resource to investigate *bHLH* family genes and to examine their potential roles in responses to cold stress. In the current study, we first carried out a genome-wide identification and analyses of *bHLH* genes in sweet orange. Based on time-course expression patterns of the *CsbHLH* genes under cold stress, we focused on *CsbHLH18*, the most cold-inducible member. Our results revealed that CsbHLH18 plays a positive role in cold tolerance, which was attributed, at least partly, to modulation of homeostasis of reactive oxygen species (ROS) by regulating antioxidant genes. Our findings shed valuable light on functional elucidation of the *bHLH* genes in sweet orange, and underpin the exploration of genes of significance for genetic manipulation.

## Materials and methods

### Plant materials and treatments

Seven-year-old sweet orange plants were grown in the open field under natural photoperiod conditions and watered twice a week. For analysis of expression patterns of the *CsbHLH* genes, uniform and healthy fresh shoots excised from the plants were placed in conical flasks containing sterile deionized water for 24 h and kept in a growth room with a 16 h light/8 h dark photoperiod at 25 °C. For the cold treatment, the flasks were kept in a low-temperature (4 °C) growth chamber and the leaves were sampled at designated time points (0, 6, 12, 24, 48, and 72 h). The collected samples were immediately frozen in liquid nitrogen and stored at –80 °C until further analyses.

### Genome-wide identification and bioinformatics analysis of *CsbHLH* genes

Putative *bHLH* genes were retrieved using the released sweet orange genome database (Xu *et al.*, 2013). Positions of the candidate genes in the chromosomes were determined using the Orange Genome Project database, while the distribution of the *CsbHLH* genes on the chromosomes was processed using MapInspect. Genomic sequences with the same start and stop sites on the same chromosome were considered as one gene, and the longest coding sequence (CDS) was retained. A gene structure map was constructed using the Gene Structure Display Server Program (Hu *et al.*, 2015). The online programs SMART (Letunic *et al.*, 2012) and Pfam were used to predict the HLH domains; genes without the conserved HLH domains were excluded. The amino acids of the bHLH domains were aligned using Clustal W (Chenna *et al.*, 2003), while WEBLOGO (Crooks *et al.*, 2004) was used to reveal the conserved motifs in the bHLH domains. Based on the conserved domains of *bHLH* genes in sweet orange and *A. thaliana* (Toledo-Ortiz *et al.*, 2003), a phylogenetic tree was constructed using the Neighbor–Joining method in MEGA5.0 (Tamura *et al.*, 2011) with 1000 bootstrap replicates.

### RNA extraction, RT–PCR, and qPCR

Total RNA was extracted from the leaves using TRIzol reagent (TaKaRa, Dalian, China) according to the manufacturer’s instructions and then reversely transcribed to cDNA using a First Strand cDNA Synthesis Kit (TaKaRa). Semi-quantitative reverse transcription–PCR (RT–PCR) analysis was carried out according to Shi *et al.* (2010) with the exception of using primers specific to the *CsbHLH* genes. Quantitative real-time RT–PCRs (qPCRs; 95 °C, 5 min; 95 °C, 10 s; 56–63 °C 30 s, 45 cycles) were conducted with the QuantStudio 7 Flex system (Applied Biosystems, USA) using the SYBR Premix ExTaq Green PCR Kit (QIAGEN, Germany). The 10 μl qPCR solutions contained 5 μl of SYBR Green PCR Master Mix, 0.25 μM of forward and reverse primers, and 100 ng of cDNA template. *Actin* and *Ubiquitin* were used as internal reference genes for sweet orange and tobacco, respectively. Relative expression levels of the examined genes were calculated using the 2^−∆∆CT^ method (Livak and Schmittgen, 2001). Four biological replicates were performed for each sample. Gene-specific primers (Supplementary Table S1) were designed using the online NCBI Primer BLAST.

### Subcellular localization analysis

The *CsbHLH18* CDS without the stop codon was cloned into the 101YFP (yellow fluorescent protein) vector at *Xba*I and *Sal*I restriction sites, under the control of the *Cauliflower mosaic virus 35S* (CaMV 35S) promoter. For transient expression analysis, the fusion construct (bHLH18–YFP) or the control vector (YFP) was co-transformed with a plasmid coding for a nuclear marker VirD2NLS fused to mCherry into tobacco (*Nicotiana benthamiana*) leaves by *Agrobacterium tumefaciens* infiltration based on a previous description (Kumar and Kirti, 2010). The infiltrated plants were placed in the dark for 24 h and grown for an additional 2 d under a 16 h light/8 h dark photoperiod. In addition, the infiltrated leaves were subjected to enzymatic digestion for isolation of mesophyll protoplasts as reported by Yoo *et al.* (2007) with slight modification. Yellow (for YFP) and red (for mCherry) signals in the epidermis and protoplasts were observed using a laser scanning confocal microscope (Leica TCS-SP8, Germany).

### Transcriptional activation analysis

The full length (FL) or three truncated fragments (F1, F2, or F3) of CsbHLH18 were PCR amplified and cloned into pGBKT7 (Clontech) to generate four constructs (pGBKT7-FL/F1/F2/F3). The fusion vectors and empty vector (pGBKT7), used as a negative control, were separately transformed into the yeast (*Saccharomyces cerevisiae*) strain AH109 harboring a MEL1 reporter. Transcriptional activation activity of the transformed yeast cells was determined after incubation at 30 °C for 3 d on SD/-Trp or SD/-Trp/-His/-Ade medium supplemented with 4 mg ml^–1^ 5-bromo-4-chloro-3-indoxyl-α-d-galacto-pyranoside (X-α-gal, Sigma-Aldrich).

### Transformation and characterization of transgenic plants

The CDS of *CsbHLH18* was amplified and ligated into the *Xba*I/*Bam*HI sites of pBI121 (Clontech) to obtain an overexpression vector. For construction of the RNAi vector, a 252 bp fragment was PCR amplified and integrated into the pHELLSGATE vector. The constructs were verified by sequencing and introduced into *A. tumefaciens* strain GV3101 by heat shock. The overexpression vector was used to transform tobacco (*N. nudicaulis*), while the RNAi vector was transformed into trifoliate orange (*Poncirus trifoliata*). Transformation of tobacco and *P. trifoliata* was carried out as previously reported (Huang *et al.*, 2013). The transformants were selected on MS (for tobacco; Murashige and Skoog, 1962) or MT (for trifoliate orange; Murashige and Tucker, 1969) medium containing 10 μg ml^–1^ kanamycin. The presence of the transgene was confirmed by genomic PCR according to a procedure reported by Gong *et al.* (2015), while expression levels of the transgene or endogenous bHLH18 in the positive lines was examined via both RT–PCR and qPCR.

### Cold tolerance assays

Seeds of transgenic tobacco lines at the T_2_ generation and the wild type (WT) were germinated on wet filter papers under a 16 h light/8 h dark photoperiod at 25 °C. Four days later, the seedlings were transferred to soil pots and kept in a growth chamber (16 h light/8 h dark photoperiod, 25 °C). For cold treatment, 2-week-old plants were shifted to a growth chamber set at 4 °C for 10 h, then at 0 °C for 8 h, and finally –2 °C for 2 h, followed by recovery at 25 °C for 15 h. In addition, 6-month-old tobacco plants were subjected to –2 °C for 3 h, and finally recovered for 12 d at room temperature. As for cold tolerance assessment of *P. trifoliata*, seedlings of 5-month-old WT and RNAi lines were exposed to –4 °C for 24 h. Plant performance was monitored before and after the cold treatment, while survival rates (only for tobacco) were calculated after the recovery. Chlorophyll fluorescence was measured using an IMAGING-PAM chlorophyll fluorimeter (under a single saturating pulse of >1800 μmol photons m^–2^ s^–1^), based on which Fv/Fm ratios were calculated using Imaging WinGegE software (Walz, Germany). The leaves were collected after cold treatment for physiological measurement and gene expression analysis.

### Physiological analyses

Electrolyte leakage (EL) was analyzed essentially following an earlier report (Dahro *et al.*, 2016). Malondialdehyde (MDA) levels, expressed as nmol mg^–1^ protein, were determined based on a thiobarbituric acid (TBA) reaction (Liu *et al.*, 2006). In brief, MDA was extracted by homogenizing 0.1 g of leaf powder in 1 ml of 20% (w/v) trichloroacetic acid (TCA), followed by centrifugation at 5000 rpm for 20 min at 4 °C. A 1 ml aliquot of the supernatant was mixed with 1 ml of 20% TCA containing 0.5% TBA (w/v) and 100 µl of 4% butylated hydroxyl toluene in ethanol (v/v). The mixture was kept for 30 min in a water bath at 95 ^o^C and then cooled down on ice for 20 min. After centrifugation at 10 000 rpm for 5 min, absorbance of the supernatant was measured at 532 nm. MDA concentration was calculated as described by Heath and Packer (1968).

For extraction of proline, antioxidant enzymes, and H_2_O_2_, ~0.1 g of each frozen sample was homogenized in 1 ml of ice-cold extraction buffer (100 mM potassium phosphate buffer, pH 7.8, containing 1% polyvinylpyrrolidone). The homogenate was centrifuged at 12 000 rpm for 10 min at 4 °C and the resultant supernatant was collected for assays of proline, H_2_O_2_, and antioxidant enzymes, namely superoxide dismutase (SOD; EC 1.15.1.1), catalase (CAT; EC 1.11.1.6), and peroxidase (POD; EC 1.11.1.7), using relevant detection kits (A107 for proline, A064-1 for H_2_O_2_, A084-3 for POD, A001-1 for SOD, and A007-1 for CAT, Nanjing Jiancheng Bioengineering Institute, Jiangsu, China) following the manufacturer’s instructions. Proline levels (µg g^–1^ FW) were monitored by reading the absorbance at 520 nm, while the absorbance of H_2_O_2_ was determined at a wavelength of 415 nm. Enzyme activity of the samples was recorded as U mg^–1^ protein. One unit of SOD activity was defined as the amount of enzyme required for 50% inhibition of nitroblue tetrazolium (NBT) as monitored at 550 nm. One unit of POD activity was defined as an increase of 0.01 min^–1^ in the absorbance at 470 nm, while one unit of CAT activity was defined as a reduction of 0.01 min^–1^ in absorbance at 240 nm from the decomposition of H_2_O_2_.

O_2_·^–^ measurement was performed according to Ma *et al.* (2016) with minor changes. In brief, 0.5 g of leaf powder was homogenized in 5 ml of ice-cold 65 mM potassium phosphate buffer (pH 7.8). After centrifugation at 10 000 rpm for 15 min at 4 ^o^C, 5 ml of the supernatant was mixed with 0.1 ml of 10 mM hydroxylamine hydrochloride and incubated for 20 min at 25 ^o^C, followed by addition of 1 ml of 58 mM *p*-aminobenzenesulfonic acid and 1 ml of 7 mM α-naphthylamine. The mixtures were incubated for 20 min at 25 ^o^C and then centrifuged at 4000 rpm for 10 min, and the absorbance of the supernatant was measured at 530 nm to calculate the O_2_·^–^ content.

Absorbance in all measurements was read on a spectrophotometer (UV-1800, Shimadzu, Japan), and total protein concentrations were analyzed using the Coomassie Brilliant Blue G-250 staining method (Bradford, 1976).

### Histochemical staining of ROS

*In situ* accumulation of H_2_O_2_ and O_2_·^–^ was examined by histochemical staining with 3,3'-diaminobenzidine (DAB) and NBT, respectively, according to Wang *et al.* (2011). Briefly, the leaves were placed in freshly prepared solutions of 1 mg ml^–1^ DAB (in 50 mM potassium phosphate, pH 3.8) or NBT (in 50 mM potassium phosphate, pH 7.8). After incubation for 12 h in the dark at room temperature, the chlorophyll was removed with 75% ethanol in a boiling water bath and the leaves were then photographed.

### Yeast one-hybrid (Y1H) assay

The promoters of *CsSOD* (Cs8g15520.1), *CsPOD* (orange1.1t02041.1), and *CsCAT* (Cs3g27290.1) were acquired by genomic PCR with specific primers using sweet orange genomic DNA as template. Eight promoter fragments (S1/2 for *CsSOD*, P1/2/3 for *CsPOD*, and C1/2/3 for *CsCAT*) were amplified and ligated into the pAbAi vector to generate the baits. mP1 is a mutated version of P1, in which the core sequence (CATTTG) of two E-box elements was mutated to GGCCGC and GATGCC, respectively, according to the method reported by Gong *et al.* (2015). The full-length *CsbHLH18* ORF was amplified and fused to the pGADT7-AD vector to create the prey. Y1H assay was performed following the manufacturer’s protocol (Clontech, USA).

### EMSA

The CsHLH18 CDS was amplified and cloned into the pDONR222 vector (Invitrogen) using the Gateway system, followed by site-specific recombination into the pHMGWA vector (Busso *et al.*, 2005). The resulting construct (HIS-CsHLH18) was expressed in *Escherichia coli* strain BL21 (DE3) cells, and the fusion protein was purified using glutathione Sepharose 4B beads (GE Healthcare, NJ, USA). EMSA was performed using the LightShift Chemiluminescent EMSA Kit (Pierce, IL, USA) according to the manufacturer’s protocol. The 39 bp biotin-labeled DNA probes containing either WT or mutated E-box elements, and unlabeled competitor DNAs were synthesized by Shanghai Sangon Biotechnology based on P1 sequences of *CsPOD*. The binding reaction was performed for 20 min at room temperature in 20 μl of reaction buffer containing 1 μl of poly(dI–dC) (1 μg μl^–1^), competitor DNA at 150, 100, or 0 nM, 17.6 μg of recombinant protein, and 1 nM biotin-labeled probe. Protein–DNA samples were separated in a 6% polyacrylamide gel.

### Transient expression assays

The CDS of *CsbHLH18* was ligated into the transient expression vector pGreen II 62-SK to generate an effector plasmid, while P1 or mP1 fragment were fused into the vector pGreen II 0800-LUC to produce the reporter plasmids (Hellens *et al.*, 2005). The effector, each of the two reporter constructs, and a helper plasmid pSoup (Vain *et al.*, 2003) were co-transformed into *A. tumefaciens* GV3101. Transient expression assay in *N. benthamiana* leaves was performed based on Espley *et al.* (2009) with minor modifications. The activities of firefly luciferase (LUC) and *Renilla* luciferase (REN) were measured using the Dual-Luciferase® Reporter Assay System (Promega, WI, USA) on an Infinite 200 Pro microplate reader (Tecan). The promoter activity was expressed as the ratio of LUC to REN.

### Statistical analysis

Cold treatment was repeated at least twice with three replicates for each line. All the data were statistically evaluated using SPSS software (SPSS Statistics); statistical differences were determined using ANOVA based on Fisher’s LSD test, taking *P*<0.05 as significantly different.

## Results

### Identification and chromosomal distribution of sweet orange *bHLH* genes

**Fig. 1.**
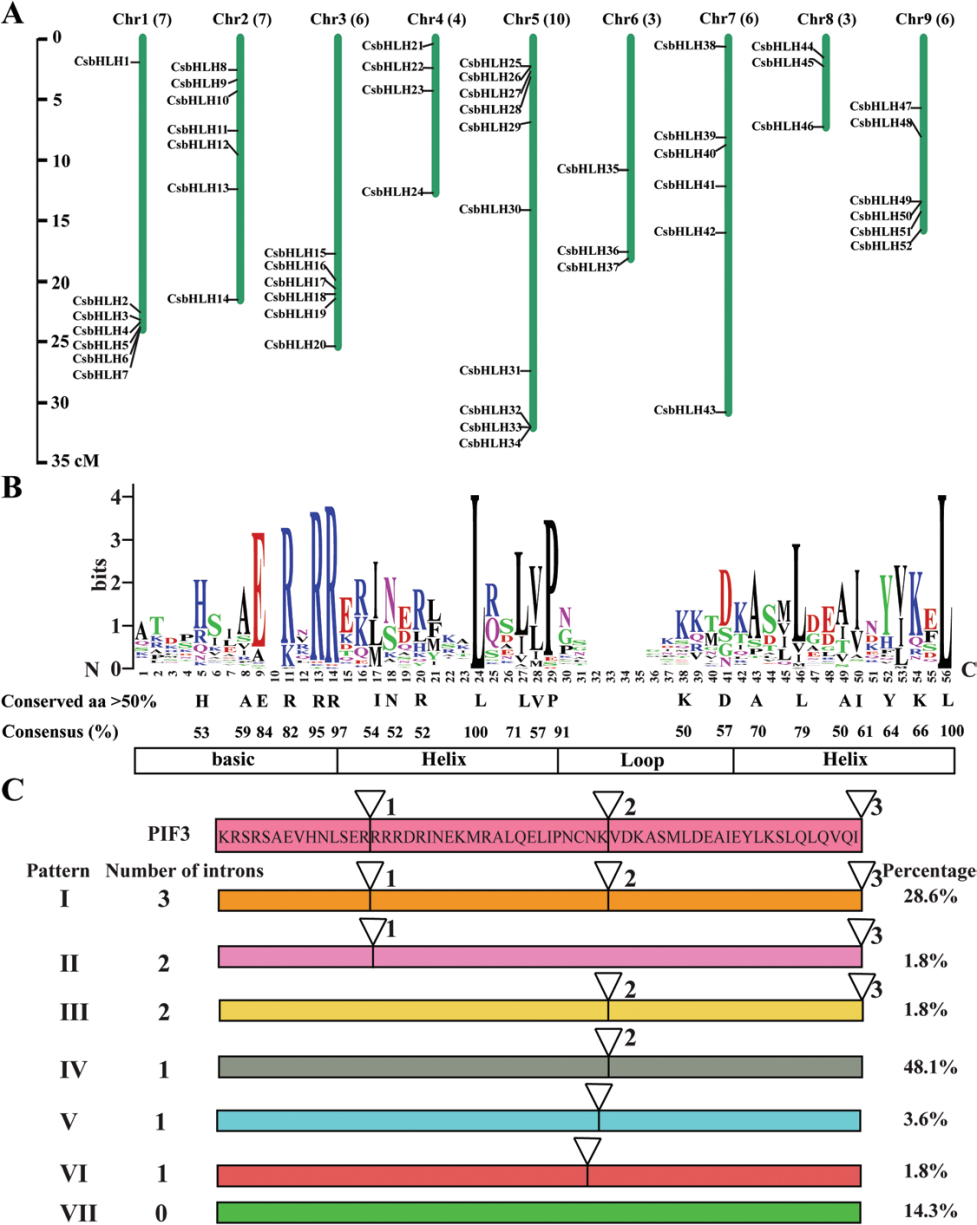
Chromosomal distribution, sequence logos, and intron number within the bHLH domains of *CsbHLH* genes. (A) Chromosomal distributions of *CsbHLH* genes. The chromosomal positions of *CsbHLH* genes are mapped according to the sweet orange genome and named from *CsbHLH1* to *CsbHLH52* based on their order in the nine chromosomes. The chromosome numbers are indicated on the top of each chromosome. (B) Sequence logos of the bHLH domains. The overall height of the stack indicates the sequence conservation of the corresponding amino acid. The upper case letters below the logo indicate >50% consensus amino acids among all CsbHLH domains. (C) Intron distribution patterns, designated as Type I–VII, within the conserved bHLH domain of *CsbHLH* genes. Intron positions are marked with inverted triangles and numbered (1–3) based on the reference gene *PIF3* from *A. thaliana*. The number and percentage of genes in each category are shown on either side of the intron patterns. (This figure is available in colour at *JXB* online.)

A total of 96 potential candidates were retrieved when ‘bHLH’ was used as a query to search the sweet orange genome database. Non-redundant sequences were obtained by removing different transcripts from the same gene, and the longest sequences were used to confirm the presence of HLH domains based on analyses by SMART. Finally, 56 genes were identified as putative bHLH members, of which 52 could be mapped to sweet orange chromosomes. They were named according to their orders in the nine chromosomes (Fig. 1A; Supplementary Table S2). The percentage of *CsbHLH* genes per chromosome varied from 0.15% on chromosome 6 to 0.37% on chromosomes 9, with an average of 0.24% (Table 1), indicating an uneven distribution of the bHLH genes. The greatest number of *CsbHLH* genes was on chromosome 5 (10 genes), followed by seven on chromosomes 1 and 2, six on chromosomes 3, 7, and 9, four on chromosome 4, and three on chromosomes 6 and 8.

**Table 1.** The distribution ratio of *CsbHLH* genes among chromosomes

Chromosomes	**Total number of genes**	**Number of *CsbHLH* genes**	**Percentage of *CsbHLH* genes in the chromosome (%**)
1	2403	7	0.29
2	2780	7	0.25
3	2509	6	0.24
4	1809	4	0.22
5	3140	10	0.32
6	1960	3	0.15
7	2926	6	0.2
8	1828	3	0.16
9	1625	6	0.37

Total numbers of genes on each of the nine sweet orange chromosome based on the NCBI database (https://www.ncbi.nlm.nih.gov/)

### Sequence features of bHLH domains

Multiple sequence alignment using the bHLH domains was carried out to understand the degree of conservation and relative frequency of corresponding amino acids at each position (Fig. 1B). We observed that 22 amino acid residues were highly conserved with at least a 50% consensus ratio; in particular, the residues Arg-13, Arg-14, Leu-24, Pro-29, and Leu-56 had a consensus ratio >90%.

It has been documented that the bHLH proteins can be generally classified into two subgroups according to their DNA binding characteristics: E-box-binding members (including G-box binders and non-G-box binders) and non-E-box-binding members (Toledo-Ortiz *et al.*, 2003). Among the CsbHLH proteins, 46 contain E-box-binding regions, including 33 G-box binders and 13 non-G-box binders (Table 2). In addition, we identified seven putative non-E-box-binding proteins that lack either or both of the two essential amino acids (Glu-12 and Arg-15) in the basic region. Of note, three CsbHLH proteins were categorized in the non-DNA-binding subgroup as they contain less than six amino acids and lack Glu-12 and Arg-15 in the basic regions.

**Table 2.** Predicted DNA binding features based on the conserved bHLH domains of CsbHLH proteins

Predicted activity	**Predicted motif**	**Numbers and percentage of proteins**
DNA binding		
E-box binding		
G-box binding	bHLH	33 (58.93%)
Non-G-box binding	bHLH	13 (23.21%)
Non-E-box binding	bHLH	7 (12.5%)
Total		53 (94.64%)
Non-DNA binding	HLH	3 (5.36%)

### Intron distribution and phylogenetic relationship of CsbHLHs

We next analyzed the intron/exon structures in the conserved bHLH domains of the *CsbHLH* genes. There are seven patterns of intron distribution, with intron number ranging from 0 to 3 (Fig. 1C). Of the CsbHLHs, 86% have introns in the conserved domains (patterns I–VI), and 80% had at least one highly conserved intron position (patterns I–IV) compared with the reference gene PIF3 from *A. thaliana*. Only 5% of the *CsbHLH* genes had different intron insertions within the conserved domains (patterns V and VI). Family members without intron insertions in their bHLH domains accounted for 14% (pattern VII). Pattern IV was the most common type (~48%), followed by pattern I (29%).

**Fig. 2.**
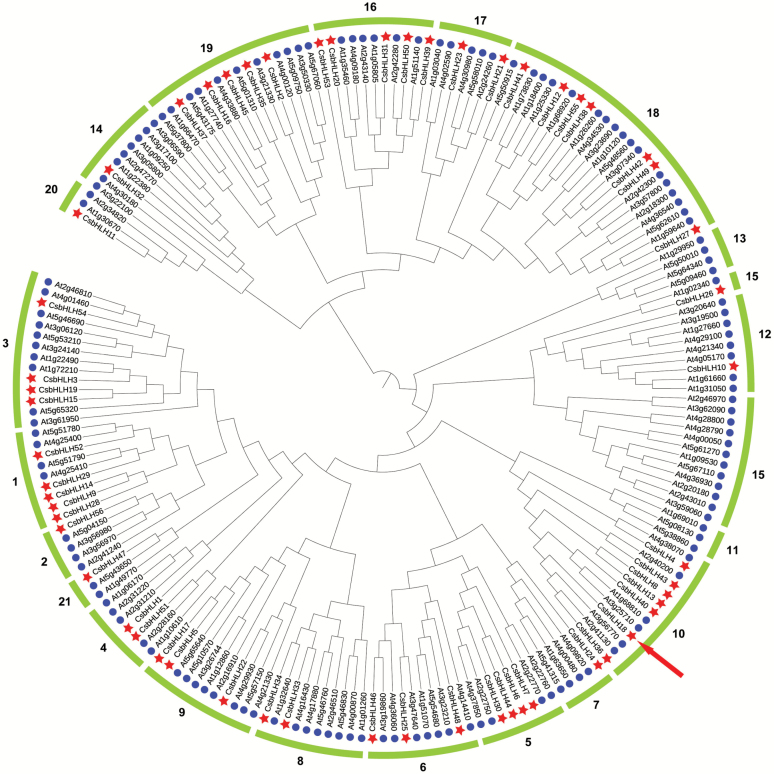
Phylogenetic tree of bHLH TFs constructed based on bHLH domains from *Arabidopsis thaliana* (circles, prefixed by At) and *Citrus sinensis* (stars, prefixed by Cs). The lines with numbers represent different subfamilies. (This figure is available in colour at *JXB* online.)

To get a better understanding of the evolutionary relationship and classification of the CsbHLH members, a Neighbor–Joining phylogenetic tree was generated based on amino acid sequences of the bHLH domains from *A. thaliana* and sweet orange (Fig. 2). The bHLH proteins can be clustered into 21 subfamilies, but subfamilies 11 and 13 were absent from sweet orange. In addition, gene structure analysis revealed that the *CsbHLH* genes from the same subgroups share similar structures (Supplementary Fig. S1).

### Expression profiles of *CsbHLHs* under cold treatments

**Fig. 3.**
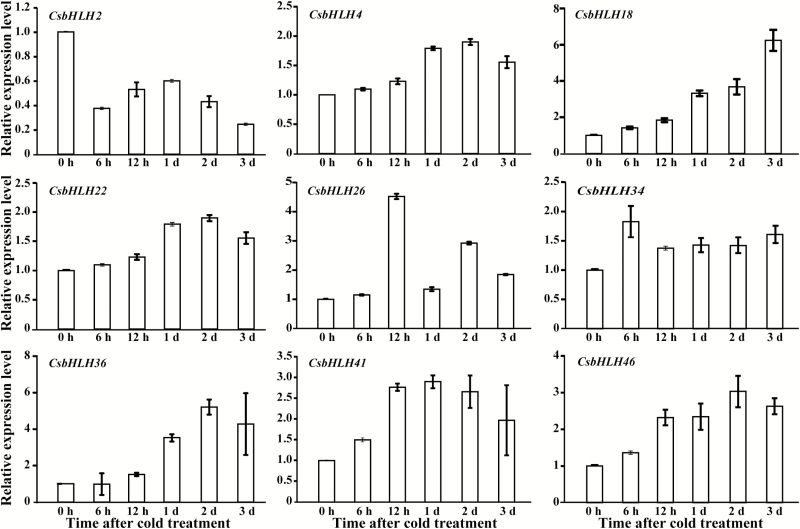
Expression patterns of *CsbHLH* genes under cold treatment. Quantitative real-time RT-PCR was carried out to examine the relative expression levels of nine *CsbHLH* genes (*CsbHLH2*, *CsbHLH4*, *CsbHLH18*, *CsbHLH22*, *CsbHLH26*, *CsbHLH34*, *CsbHLH36*, *CsbHLH41*, and *CsbHLH46*) in response to cold stress. *Actin* was used as an internal control. Transcript levels of the tested genes were calculated using the 2^−∆∆CT^ method. Error bars indicated SDs, and each of the samples had three technical replicates.

To gain insights into the potential functions of the *CsbHLH* genes, we analyzed the expression patterns of all 56 genes under cold stress by semi-quantitative RT–PCR. Sixteen genes (*CsbHLH1*, *3*, *6*, *7*, *11*, *12*, *17*, *19*, *28*, *29*, *30*, *32*, *45*, *50*, *51*, and *56*) were not expressed at detectable levels (Supplementary Fig. S2), while a total of 40 *CsbHLH* genes showed alteration of transcript levels in response to the cold treatment. The expression patterns of the genes varied among different members. *CsbHLH2*, *CsbHLH5*, *CsbHLH9*, *CsbHLH15*, *CsbHLH16*, *CsbHLH21*, *CsbHLH24*, *CsbHLH27*, *CsbHLH33*, *CsbHLH35*, *CsbHLH37*, *CsbHLH38*, *CsbHLH39*, *CsbHLH42*, *CsbHLH44*, *CsbHLH47*, *CsbHLH48*, and *CsbHLH54* were down-regulated by cold, while *CsbHLH4*, *CsbHLH18*, *CsbHLH22*, *CsbHLH26*, *CsbHLH34*, *CsbHLH36*, *CsbHLH41*, *CsbHLH43*, *CsbHLH46*, and *CsbHLH49* showed increased expression levels during the cold treatments. To confirm the RT–PCR results, nine up-regulated genes were further analyzed using qPCR, which largely validated the semi-quantitative PCR results, indicating that these genes are truly responsive to the cold stress (Fig. 3). Of the cold-responsive genes, transcript levels of *CsbHLH18* were drastically and progressively elevated by the cold stress. Therefore, it was chosen for further functional analysis.

### CsbHLH18 localizes to the nucleus

**Fig. 4.**
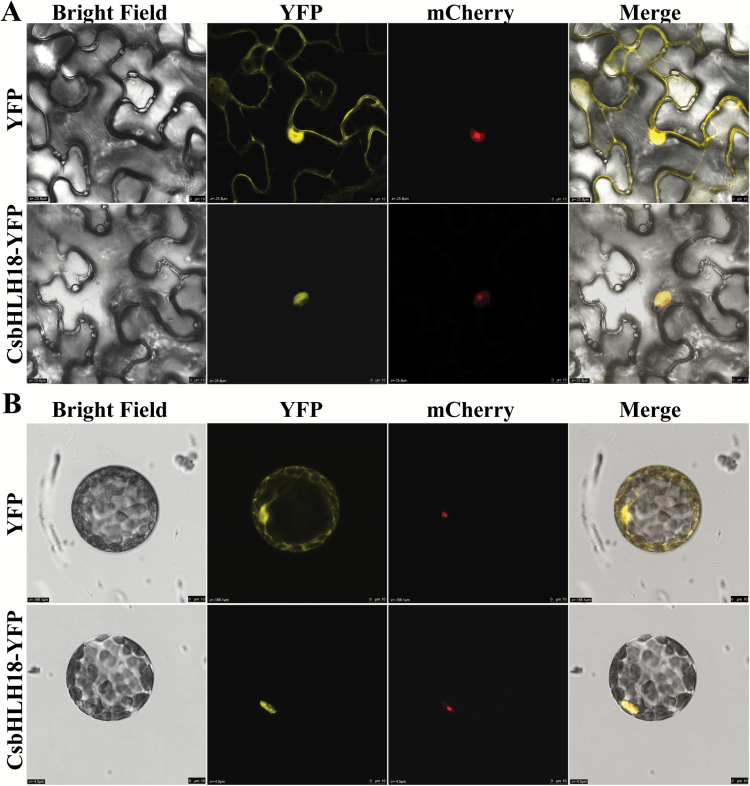
Subcellular localization of CsbHLH18 protein. (A, B) Confocal images showing yellow (for YFP) and red (for mCherry) fluorescence signals in epidermal cells (A) and protoplasts (B) isolated from the infiltrated tobacco (*N. benthamiana*) leaves. Tobacco leaves were agroinfiltrated with YFP (yellow fluorescent protein) empty control or CsbHLH18–YFP fusion protein, along with a nuclear marker gene VirD2NLS fused to mCherry. Scale bars=10 µm

To examine the subcellular distribution of the CsbHLH18 protein, the fusion vector (CsbHLH18–YFP) and the control vector (YFP) were transiently expressed in tobacco leaves. Confocal imaging of the epidermis showed that the YFP alone was detected throughout the entire cell, while the CsbHLH18–YFP fusion protein localized exclusively in the nucleus (Fig. 4A). To verify the subcellular localization using epidermis, YFP signals in tobacco protoplasts were also detected. As expected, YFP protein was observed in both the nucleus and cytoplasm in the control, whereas CsbHLH18–YFP was only detected in the nucleus (Fig. 4B). Localization in the nucleus was confirmed using co-transformation of a nucleus marker gene fused to mCherry in both epidermis and protoplasts. These results indicate that CsbHLH18 is a nuclear protein.

### CsbHLH18 possesses transcriptional activation activity

**Fig. 5.**
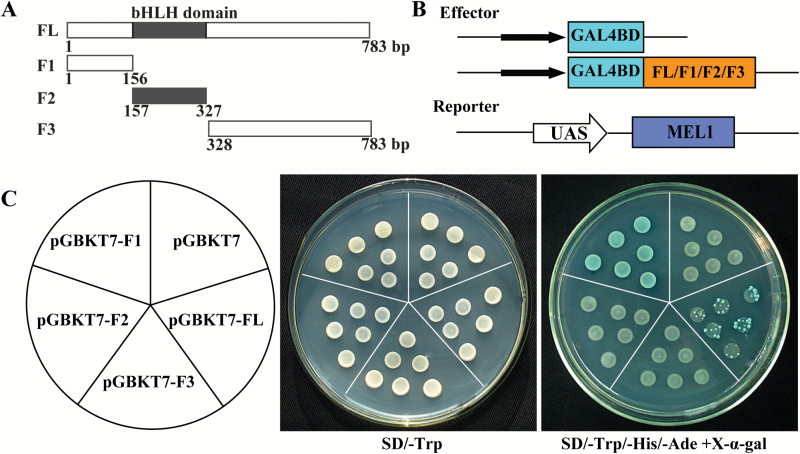
Transcriptional activity assay of CsbHLH18. (A) Schematic diagrams of the full length (FL) and three truncated fragments (F1, F2, and F3) of CsbHLH18 used for constructing vectors. (B) Effectors and reporter used for transcriptional activation activity assay. The FL and the three fragments (F1, F2, and F3) of CsbHLH18 were introduced into the pGBKT7 vector, generating four effectors (pGBKT7-FL/F1/F2/F3). (C) Growth of yeast cells (strain AH109) transformed with each of the four vectors or pGBKT7 empty vector (used as a negative control) on SD/-Trp or SD/-Trp/-His/-Ade with addition of X-α-gal. The layout of the effectors is shown in the pie chart on the left. (This figure is available in colour at *JXB* online.)

To determine whether CsbHLH18 has transcriptional activation activity, we used a heterologous yeast expression system. The FL or three truncated fragments (F1, F2, and F3) of CsbHLH18 were fused to pGBKT7 to generate four effectors, which were separately transferred into the yeast containing a MEL1 reporter (Fig. 5A, B). All of the yeast cells grew well on the SD medium lacking tryptophan (SD/-Trp), whereas only yeast cells transformed with the effectors containing FL and F1 grew and displayed GAL4 activity on the medium supplemented with X-α-gal (Fig. 5C). These results demonstrate that CsbHLH18 has transcriptional activation activity and the F1 region is necessary for the transactivation.

### Overexpression of *CsbHLH18* enhances cold tolerance in transgenic tobacco

**Fig. 6.**
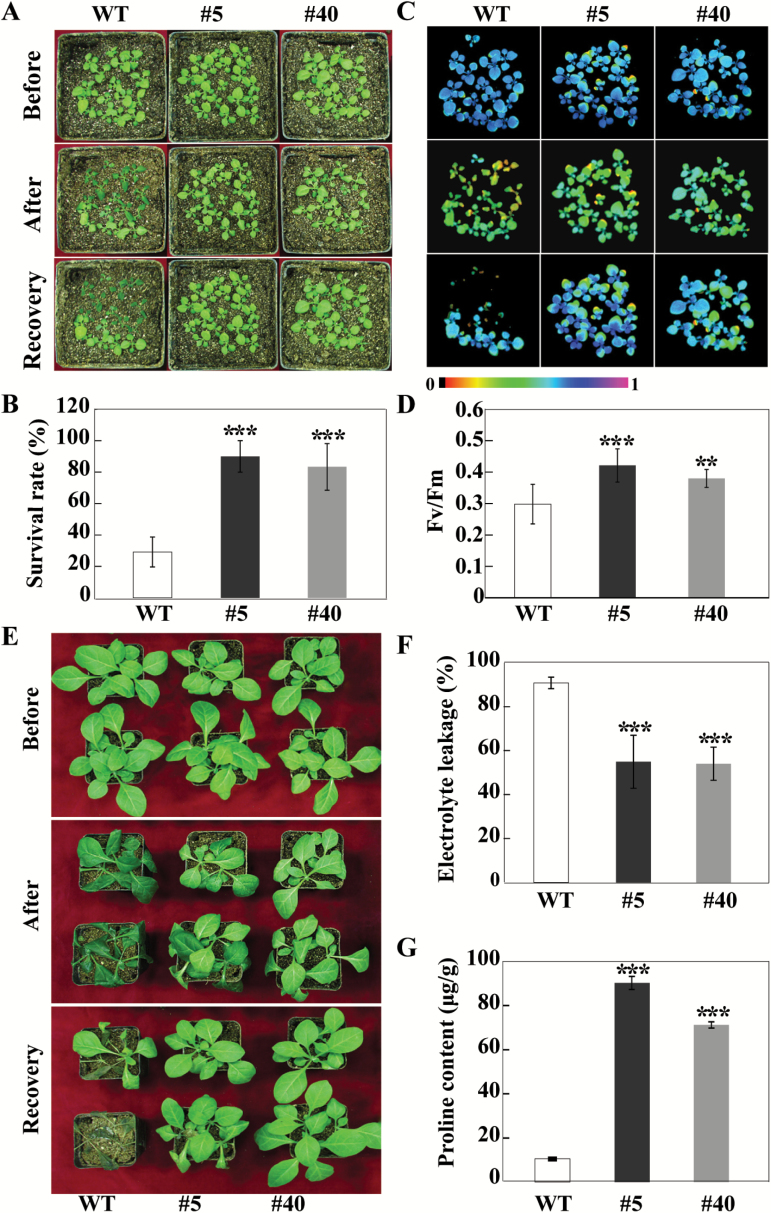
Overexpression of *CsbHLH18* confers enhanced cold tolerance to transgenic tobacco. (A) Phenotypes of 2-week-old plants of transgenic lines (#5 and #40) and the wild type (WT) before and after freezing treatment (–2 ^o^C for 2 h) and 15 h after growth recovery at ambient temperature. (B) Survival rates of WT and transgenic plants after the growth recovery, which is calculated based on the ratio of recovered plants to the total number of tested plants. (C, D) Chlorophyll fluorescence imaging (C) and Fv/Fm ratios (D) of transgenic lines and the WT after the freezing treatment. (E) Phenotypes of 6-week-old plants of transgenic lines and the WT before and after freezing treatment, followed by growth recovery for 12 d at ambient temperature. (F, G) Electrolyte leakage (F) and proline content (G) of transgenic lines and the WT, measured after the freezing treatment. Error bars represent SDs for three independent replicates. Asterisks indicate significant differences between the WT and the transgenic lines (**P<0.01, ***P<0.001).

To further investigate the function of *CsbHLH18* in cold tolerance, we generated transgenic tobacco plants overexpressing *CsbHLH18*. Two transgenic lines (#5 and #40), which had different overexpression levels of *CsbHLH18* (Supplementary Fig. S3), were selected for cold tolerance assay. No phenotypic differences were observed between the WT and transgenic lines without cold stress. When 2-week-old plants were subjected to freezing treatment, the WT exhibited a more severe water-soaking phenotype compared with the transgenic lines. After growth recovery at ambient temperature for 15 h, 80–90% of the transgenic plants recovered, whereas the survival rate of the WT was only 29% (Fig. 6A, B). In addition, leaf chlorophyll fluorescence imaging of the WT was prominently repressed relative to the transgenic lines after the freezing treatment and after the recovery. Meanwhile,The Fv/Fm ratio of the WT was significantly lower than those of the transgenic plants (Fig. 6C, D).

Likewise, when 6-week-old seedlings were exposed to freezing treatment, the leaves of transgenic lines showed a less severe water-soaking phenotype compared with the WT. Most of the transgenic plants recovered after being returned to room temperature for 12 d, while only a few WT plants recovered (Fig. 6E). EL was significantly higher in the WT (90%) than in the transgenic lines (Fig. 6F). We also measured levels of proline, which has been reported to act as an important stress indicator (Szabados and Savouré, 2010). The transgenic lines had significantly higher proline content than the WT after the cold treatment (Fig. 6G). These results indicate that overexpression of *CsbHLH18* led to enhanced cold tolerance in the transgenic plants.

### The overexpressing plants accumulate less ROS

**Fig. 7.**
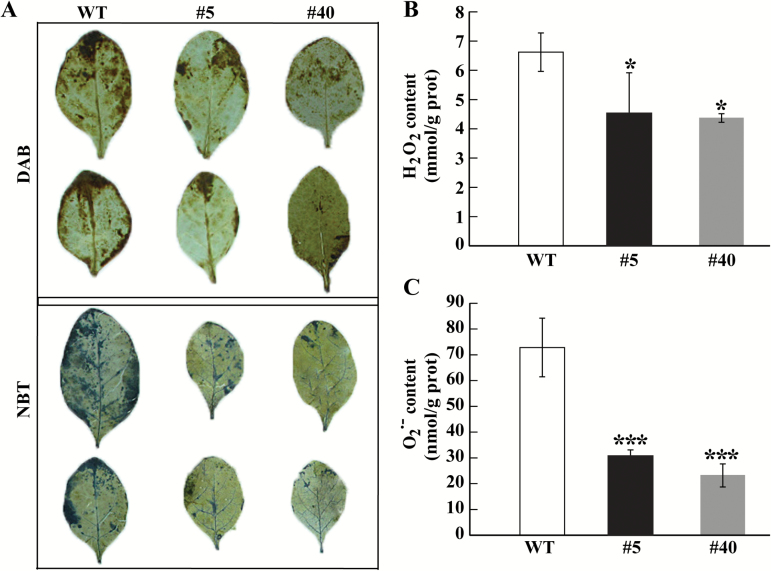
Reactive oxygen species (ROS) levels in wild-type (WT) plants and transgenic lines. (A) Histochemical staining with DAB (upper panels) and NBT (bottom panels) for detection of *in situ* accumulation of H_2_O_2_ and O_2_·^–^, respectively, in the transgenic lines (#5 and #40) and the WT after cold treatment. (B, C) Quantitative measurement of H_2_O_2_ (B) and O_2_·^–^ (C) in the transgenic and WT plants. Error bars represent SDs for three independent replicates. Asterisks indicate significant differences between the WT and transgenic lines (*P<0.05, ***P<0.001). (This figure is available in colour at *JXB* online.)

ROS are known to be responsible for causing oxidative stress that negatively influences cell integrity (Choudhury *et al.*, 2017). Since the transgenic lines exhibited greater cold tolerance than the WT, we examined the accumulation of two major ROS, H_2_O_2_ and O_2_·^–^. Histochemical staining showed that the WT leaves showed deeper staining by DAB and NBT than those of the transgenic lines (Fig. 7A). Histochemical detection of ROS was further confirmed by quantitative measurements, which showed that cellular levels of H_2_O_2_ and O_2_·^–^ were lower in the transgenic lines than in the WT (Fig. 7B, C). Both histochemical staining and measurement demonstrate that the transgenic lines accumulated lower levels of ROS in response to cold stress.

### The transgenic lines exhibit higher activities and expression levels of antioxidant enzymes and genes

**Fig. 8.**
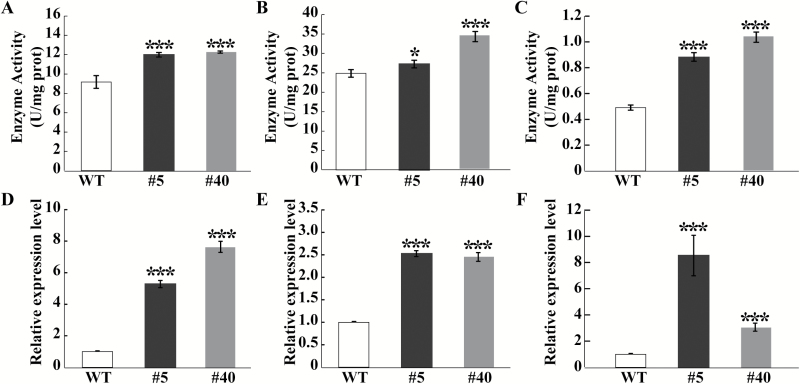
Activities and gene expression levels of antioxidant enzymes in the transgenic lines and the WT. (A–C) Activities of POD (A), SOD (B), and CAT (C) in the transgenic lines and WT measured after cold treatment. (D–F) Expression levels of *NtPOD* (D), *NtSOD* (E), and *NtCAT* (F) in the transgenic lines and the WT. *Ubiquitin* was used as an internal reference control, and transcript levels of the tested genes were calculated using the 2^−∆∆CT^ method. Error bars represent SDs for three independent replicates. Asterisks indicate significant differences between the WT and transgenic lines (*P<0.05, ***P<0.001).

Antioxidant enzymes play crucial roles in detoxification of ROS and contribute to ROS scavenging under abiotic stresses (Miller *et al.*, 2010; Huang *et al.*, 2013; Zhang *et al.*, 2016). Activities of the antioxidant enzymes POD, SOD, and CAT were thus measured after the cold treatment. Activities of all three enzymes were significantly higher in the transgenic plants than in the WT (Fig. 8A–C), which is consistent with the lower ROS accumulation in the former lines.

To further understand molecular mechanisms underlying the enhanced cold tolerance by overexpressing *CsbHLH18*, mRNA abundance of the antioxidant genes *NtSOD*, *NtPOD*, and *NtCAT* in the WT and transgenic lines was analyzed using qPCR. Transcript levels of the three tested genes were higher in the transgenic lines than in the WT (Fig. 8D–F), indicating that overexpression of *CsbHLH18* led to an up-regulation of the antioxidant genes.

### Silencing of *bHLH18* in trifoliate orange leads to elevated cold susceptibility and excessive accumulation of ROS

**Fig. 9.**
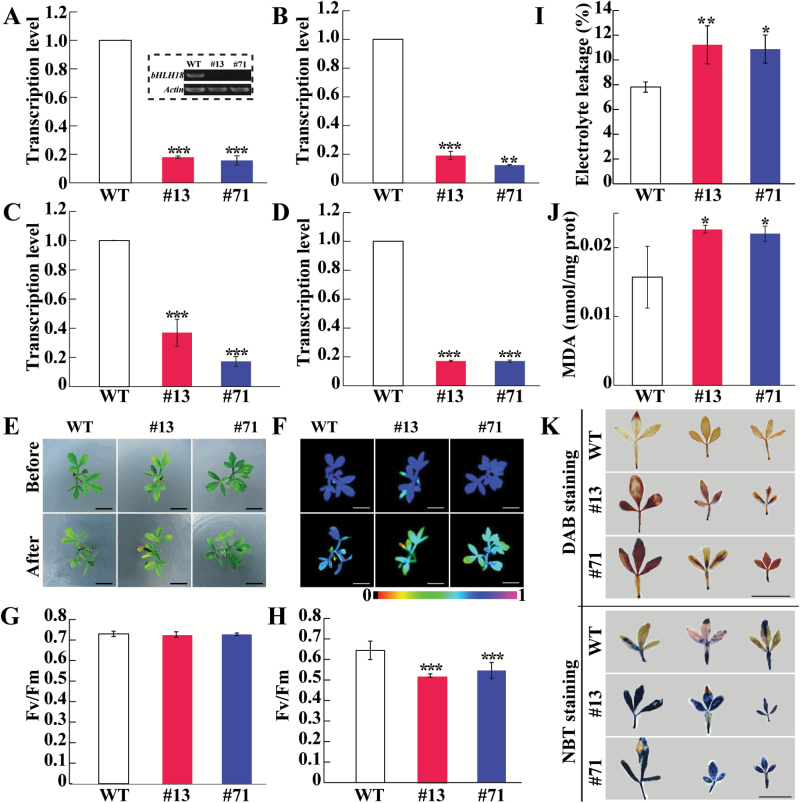
Silencing of *bHLH18* leads to increased cold sensitivity in trifoliate orange. (A) Expression levels of *bHLH18* in the two RNAi lines (#13 and #71) and the wild type (WT) based on qPCR and RT–PCR (inset image) analyses. *Actin* was used as an internal control. (B–D) Transcript levels of trifoliate orange *CAT* (B), *SOD* (C), and *POD* (D) in the RNAi lines and the WT. (E, F) Phenotype (E) and fluorescence imaging (F) of WT and RNAi lines before and after freezing treatment. (G, H) Fv/Fm ratios of the WT and RNAi lines before (G) and after (H) freezing treatment. (I–K) Electrolyte leakage (I), MDA levels (J), and *in situ* accumulation of H_2_O_2_ and O_2_·^–^ (K) in the RNAi lines and the WT. Scale bars=1 cm. Asterisks indicate significant differences between the WT and the RNAi lines (*P<0.05, **P<0.01, ***P<0.001). (This figure is available in colour at *JXB* online.)

To further elucidate the role of *bHLH18* in cold tolerance, we attempted to knock down the bHLH18 of trifoliate orange (*P. trifoliata*) using RNAi. We selected trifoliate orange for this study for the following reasons. First, trifoliate orange is closely related to sweet orange; *CsbHLH18* shares >98% nucleotide sequence identity with its counterpart gene of trifoliate orange (Supplementary Fig. S4). Secondly, it is easy to obtain seeds for production of *in vitro* seedlings so as to acquire enough shoot segments for genetic transformation. We obtained a number of positive transgenic lines by *Agrobacterium*-mediated transformation of shoot segments (Supplementary Fig. S5). RT–PCR and qPCR analyses indicated that expression levels of *bHLH18* were dramatically decreased in two putative RNAi lines (#13 and #71) compared with the WT (Fig. 9A), indicating that *bHLH18* was successfully silenced. We then measured transcript levels of the antioxidant genes *CAT*, *SOD*, and *POD* in the RNAi lines, and found that the three genes were prominently down-regulated relative to those of the WT (Fig. 9B–D). The RNAi lines were morphologically similar to the WT under normal growth conditions. However, when subjected to freezing treatment at –4 °C for 24 h, the two RNAi lines displayed more severe leaf wilting (Fig. 9E). In the absence of stress, no difference in chlorophyll fluorescence imaging and Fv/Fm ratios was observed between the tested lines, whereas the RNAi lines displayed a noticeable reduction of chlorophyll fluorescence and Fv/Fm ratios relative to the WT upon exposure to cold stress (Fig. 9F–H). In addition, levels of EL and MDA were significantly higher in the RNAi plants than in the WT (Fig. 9I–J). Meanwhile, histochemical staining with DAB and NBT showed that the RNAi lines accumulated a greater amount of H_2_O_2_ and O_2_·^–^, respectively, than did the WT (Fig. 9K). These results indicate that silencing of *bHLH18* by RNAi elevated cold sensitivity in trifoliate orange.

### CsbHLH18 directly and specifically binds to and activates the promoter of *CsPOD*

**Fig. 10.**
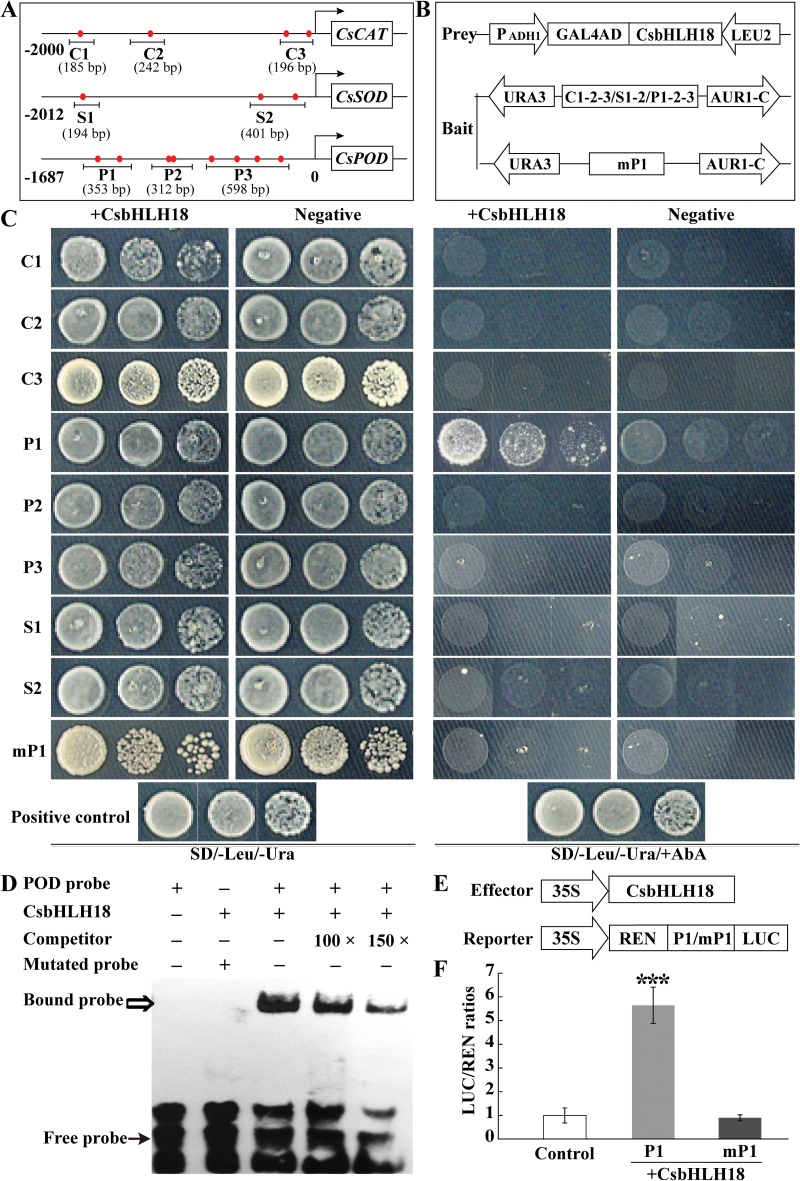
CsbHLH18 binds to and activates the promoter of *CsPOD*. (A) Schematic diagrams of the promoters of *CsCAT*, *CsSOD*, and *CsPOD*, in which the E-box elements are denoted using red circles. The segments marked with C1/2/3, P1/2/3, and S1/2 represent the promoter fragments used in the yeast one-hybrid (Y1H) assay. (B) The prey and bait vectors used for Y1H assay. mP1 is a mutated version of P1, in which the two E-box elements were mutated by PCR. (C) Growth of yeast cells of positive control (p53-AbAi+pGAD-p53), negative control (bait + pGADT7), and co-transformants (bait+prey) on SD/-Leu/-Ura medium supplemented without (left panels) or with (right panels) AbA. (D) EMSA assay using the affinity-purified fusion protein HIS-CsbHLH18 incubated with biotin-labeled probe containing the wild-type or mutated E-box element, with or without unlabeled competitor DNA. The bound DNA–protein complex is shown by an open arrow, while the solid arrow indicates the free probe. (E) Schematic diagrams of effector and reporter constructs used for transient expression assay. (F) Transient expression assay of the promoter activity using the tobacco protoplast system, based on the LUC/REN ratios. LUC/REN of the control, in the absence of the effector, was considered as 1. Asterisks indicate that the value is significantly different from that of the control (***P<0.001).

As the antioxidant genes were up-regulated in the overexpressing lines but down-regulated in the RNAi lines, we speculate that the antioxidant genes may be regulated by CsbHLH18. To verify this assumption, we obtained the promoter sequences of *CsCAT* (Cs3g27290.1), *CsPOD* (orange1.1t02041.1), and *CsSOD* (Cs8g15520.1), and found that there were four, three, and eight E-box elements on the promoters of *CsCAT*, *CsSOD*, and *CsPOD*, respectively (Fig. 10A). Interactions between CsbHLH18 and the promoters were investigated by Y1H assay. To this end, full-length cDNA of CsbHLH18 was fused with the GAL4AD to generate the prey vector, while bait vectors were constructed using eight partial fragments containing the E-box elements from the three gene promoters, three for *CsCAT*, two for *CsSOD*, and three for *CsPOD* (Fig. 10B). Y1H assay indicated that all the yeast cells grew well on SD/-Leu/-Ura medium, whereas only the positive control and yeast cells transformed with the effector and the P1 bait grew normally on the medium supplemented with Aureobasidin A (AbA). However, when the two E-box elements of the P1 fragment were mutated from CATTTG to GGCCGC and GATGCC, growth of the yeast cells was completely inhibited (Fig. 10C), indicating that CsbHLH18 interacted with the P1 region of the *CsPOD* promoter.

In order to further confirm the Y1H result, EMSA was conducted using HIS-CsbHLH18 fusion protein. There was no band shift when only the labeled probe was added to the reaction, whereas a DNA-binding band was detected when the purified fusion protein was incubated with the probe containing the WT E-box. Addition of a 100- or 150-fold excess of unlabeled competitor DNA in the reaction could compete with the labeled DNA for binding with the protein in a dosage-dependent manner. In addition, band shift was completely abolished when the E-box element in the probe was mutated (Fig. 10D). Y1H assay and EMSA indicate that CsbHLH18 directly and specifically binds to the promoter of *CsPOD.*

To further investigate the interaction between CsbHLH18 and the *CsPOD* promoter, a transient expression assay was performed using CsbHLH18 for creating the effector vector and P1 and mP1 fragments for generating the reporter vectors (Fig. 10E). The LUC/REN ratio of tobacco protoplasts co-transformed with the effector and the P1 reporter containing the WT E-box was drastically elevated relative to the control. In contrast, when the E-box element was mutated in the mP1 reporter, the LUC/REN ratio was returned to the control level (Fig. 10F). These results demonstrate that CsbHLH18 could activate the promoter of *CsPOD*.

## Discussion

Many plant TF families that participate in stress responses have been identified and characterized, such as the MYB, WRKY, CBF/DREB, and bZIPs. However, relatively little is known about the role of bHLH proteins in stress response when compared with the abundant information garnered from other families of TFs (Huang *et al.*, 2013). A genome-wide exploration of bHLH proteins in any species, and functional characterization of some of the family members, can therefore provide a better understanding of this TF family in stress signaling pathways.

In this study, 56 non-redundant sweet orange bHLH genes were identified from the available sweet orange database. It has to be mentioned that the number of bHLH proteins in sweet orange is smaller in comparison with that in other plants. Previous studies have revealed the presence of 147 *bHLH* genes in *A. thaliana* (Toledo-Ortiz *et al.*, 2003), 167 in rice (Li *et al.*, 2006), and 117 in sacred lotus (Hudson and Hudson, 2014). The exact explanation is lacking for this phenomenon at this stage. One possibility is due to fewer recent whole-genome duplications in sweet orange, which has been previously reported in earlier studies on other TFs (Hudson and Hudson, 2014; Hou *et al.*, 2014).

Chromosomal distribution analysis indicates that the *CsbHLH* genes were unevenly distributed among the nine chromosomes, whereas four members could not be definitely placed in this study. Currently, ~87% of the sweet orange genome has been published; therefore, it is possible that availability of a more detailed genome sequence in the future may help to locate the four genes in the exact chromosomes. Alignment of all the bHLH domains, which are necessary for the formation of homodimers or heterodimers, showed that the Leu-24 and Leu-56 residues in the helix regions were completely conserved, compared with 99% and 97% in tomato (Sun *et al.*, 2015). Dimerization could involve interactions between the bHLH proteins themselves, but also between bHLH proteins and other TFs. The heterodimer partners include R2R3-MYBs, BAR1-BES1, and AP2s (Goff *et al.*, 1992; Yin *et al.*, 2005; Dubos *et al.*, 2008; Chandler *et al.*, 2009). This type of dimerization has been previously shown to change or expand the diversity of intermolecular interactions and create new functions by recognizing new DNA binding sites (Heim *et al.*, 2003; Toledo-Ortiz *et al.*, 2003).

Gene expression profiles can provide important clues to understand their potential biological functions. It has been suggested that genes induced by a given abiotic stress may play a positive role in modulation of tolerance to this stress (J. Li *et al.*, 2017). To identify members that are induced by cold stress, the transcriptional patterns of CsbHLHs were investigated. Based on the strong and continuous induction of *CsbHLH18* in response to cold, we generated tobacco overexpressing plants, and the transgenic lines showed enhanced tolerance to cold stress when compared with the WT plants. On the other hand, knockdown of the *bHLH18* counterpart in trifoliate orange led to enhanced sensitivity of the RNAi lines. These results suggest that CsbHLH18 is a positive regulator of the tolerance to cold stress. It is worth mentioning that the homologous gene of *CsbHLH18* in Arabidopsis or other plants has not yet been studied in relation to cold stress, although a few bHLH TFs have been proven to play important roles in abiotic stresses (Seo *et al.*, 2011; Huang *et al.*, 2013). Hence, our findings provide a new avenue to understand the implication of *bHLH18* in cold tolerance and offer a new candidate gene with potential for genetic engineering in an effort to improve abiotic stress tolerance.

Cold stress leads to a plethora of physiological effects that may be detrimental to plant cells, one of which is accumulation of ROS that are toxic molecules causing oxidative damages to cellular components, including proteins, lipids, and DNA (Miller *et al.*, 2010). ROS may be maintained at a low level under favorable growth conditions, but are dramatically elevated when plants are challenged by abiotic stresses, leading to ROS-associated injuries. It is thus conceivable that the levels of ROS can be considered as an indicator of the magnitude of stress severity and stress tolerance, and a lower level of ROS following stress exposure is generally regarded as better tolerance. Under this scenario, the ROS level is always used for examining the difference in stress tolerance capacity between various plants. In this study, we found that the transgenic overexpressing lines accumulated prominently less H_2_O_2_ and O_2_·^–^ compared with the WT after the cold treatment, as revealed by both histochemical staining and quantitative measurement. Of note, the lower ROS levels in the transgenic lines are consistent with their better growth phenotype and less serious membrane damage (indicated by lower EL), implying that accumulation of lower ROS levels constitutes a physiological mechanism partly, if not fully, underlying *CsbHLH18*-mediated cold tolerance in the transgenic lines. This is further supported by concurrent observation of a noticeable elevation of ROS levels and enhanced cold susceptibility in the RNAi lines with knockdown of trifoliate orange *bHLH18*. It is well known that the ROS homeostasis during stress is largely dependent on the balance between ROS generation and scavenging (Miller *et al.*, 2010; Huang *et al.*, 2013). ROS-scavenging enzymes, such as POD, SOD, and CAT, are indispensable for ROS detoxification so that plants can combat the ROS-associated cellular damage and maintain better survival under stressful conditions (Miller *et al.*, 2010). Herein, the transgenic lines displayed significantly higher antioxidant enzyme activities than the WT under cold conditions, indicating that the transgenic plants may have a more powerful ROS-scavenging machinery. The greater enzyme activities may explain the lower accumulation of ROS in the transgenic lines. On the other hand, transcript levels of all three antioxidant genes were considerably suppressed in the RNAi lines, consistent with the increase of ROS levels. We thus hypothesize that bHLH18 functions in cold tolerance by controlling ROS accumulation via modulation of the antioxidant-scavenging machinery.

It is known that the bHLHs can function in the transcriptional regulation network by binding to the E-box elements within the prompter region of their target genes (Huang *et al.*, 2013). For example, MYC2 showed a specific association with the E-box of the *PLETHORA* gene during jasmonic acid-mediated modulation of the root stem cell niche in Arabidoposis (Chen *et al.*, 2011). Furthermore, AtbHLH122 was reported to play a role in abiotic stress tolerance via repressing the expression of *CYP707A3* by binding to the E-box elements in its promoter (Liu *et al.*, 2014). In the present study, the fact that the three antioxidant genes were noticeably up-regulated in the overexpressing lines but down-regulated in the RNAi lines seems to suggest that they may serve as targets of CsbHLH18. Surprisingly, despite the existence of E-box elements in the promoters of the three antioxidant genes, CsbHLH18 could only bind to and activate the promoter of *CsPOD*. This finding indicates that *CsPOD* is a direct target gene of CsbHLH18. Nevertheless, the absence of interaction between CsbHLH18 and promoters of *CsSOD* and *CsCAT* genes is intriguing as their transcript levels were altered in the same manner as that of *CsPOD* in the transgenic plants. One of the reasons for this phenomenon is the difference in the nucleotides surrounding the E-box elements, which has been reported to influence the three-dimensional structure of DNA-binding sites (Gordân *et al.*, 2013). Another possibility is that CsbHLH18 is not directly related to transcriptional control of these two genes, which are otherwise regulated by other unidentified TFs that are under the control of CsbHLH18, thus constituting a complex regulatory cascade; this speculation needs to be verified in the future. It is worth mentioning that such a phenomenon has been previously reported for other TFs. For example, Jiang *et al.* (2012) showed that overexpression of *AtWKRY57* up-regulated *RD29A*, *NCED3*, and *ABA3*, but only two of these genes (*RD29A* and *NCED3*) were confirmed to be directly regulated by AtWRKY57.

To conclude, we identified 56 bHLH proteins in sweet orange using the released genome database. Most of the TFs are responsive to cold treatment, of which *CsbHLH18* was particularly induced. Overexpression of *CsbHLH18* led to enhanced cold tolerance, while knock-down of *bHLH18* in trifoliate orange promoted cold susceptibility. CsbHLH18-mediated cold tolerance might be due, at least in part, to modulation of the antioxidant system by regulating, directly or indirectly, the antioxidant genes. Our study provides new insight into the physiological mechanism and regulatory function of the bHLH family members and also provides valuable knowledge to understand the plant cold response.

## Supplementary data

Supplementary data are available at *JXB* online.

Fig. S1. Gene structures of the *CsbHLH* genes.

Fig. S2. Expression patterns of *CsbHLH* genes in response to cold stress, as analyzed by RT–PCR.

Fig. S3. Generation and molecular identification of transgenic tobacco plants overexpressing *CsbHLH18*.

Fig. S4. Alignment of the nucleotide sequences of bHLH18 from sweet orange and trifoliate orange.

Fig. S5. Transformation and molecular characterization of trifoliate orange expressing an RNAi vector.

Table S1. Primers used in this study.

Table S2. The list of CsbHLH members identified in the sweet orange genome.

Supplementary Data
