# Supplementary material for: The transcription factor CsbHLH18 of sweet orange functions in modulation of cold tolerance and homeostasis of reactive oxygen species by regulating the antioxidant gene
Source: J Exp Bot. 2018 Feb 21;69(10):2677–92. doi: 10.1093/jxb/ery065 (PMC5920331; doi:10.1093/jxb/ery065)
Supplement: Supplementary Data [file ery065_suppl_supplementary_data.pdf]

Supplementary data for

The transcription factor CsbHLH18 of sweet orange (*Citrus sinensis*) functions in modulation of cold tolerance and reactive oxygen species homeostasis by regulating the antioxidant gene

Jingjing Geng, Ji-Hong Liu*

Key Laboratory of Horticultural Plant Biology (MOE),

College of Horticulture and Forestry Sciences,

Huazhong Agricultural University,

Wuhan, 430070

China

Supplementary Table S1. Primers used in this study.

Genes (full length or fragments)	Forward Primer (5'-3')	Reverse Primer (5'-3')	Used for
CsbHLH18	GCTCTAGAATGTTTCCGTTTCAAAGTCACC	CGGGATCCCTAATCACGATGATCACATTCG	Transgene overexpression
CsbHLH18	GCTCTAGAATGTTTCCGTTTCAAAGTCACC	ACGCGTCGACATCACGATGATCACATTCG	Subcellular localization
CsbHLH18	CGGAATTCATGTTTCCGTTTCAAAGTCACC	ACGCGTCGACATCACGATGATCACATTCG	Transcriptional activation assay
CsbHLH18-F1	CGGAATTCATGTTTCCGTTTCAAAGTCACC	ACGCGTCGACTGATTTCTTCTCCACCTTTG	
CsbHLH18-F2	CGGAATTCACTTCTGATGCATGCAAGAG	ACGCGTCGACCGTAGCTTGGCTTCTCAG	
CsbHLH18-F3	CGGAATTCGACGTGGCGGAACGTGATT	ACGCGTCGACATCACGATGATCACATTCG	
CaMV35S-CsbHLH18	TCTCCACTGACGTAAGGGATGACGC	CGGGATCCCTAATCACGATGATCACATTCG	Confirmation of transgenic lines
NPPII	AGACAATCGGCTGCTCTGAT	TCATTTCGAACCCCAGAGTC	
CsbHLH18	GCCATGGAGGCCAGTGAATTCATGTTTCCGTTTCAAAGTCACC	CAGCTCGAGCTCGATGGATCCCTAATCACGATGATCACATTCG	Y1H assay

CsSOD-S1	CTTGAATTCGAGCTCGGTACCGAAAAGCAACCCCTGAAGATC	ATACAGAGCACATGCCTCGAGTGGACAAGGGACTCGACGTAG	
CsSOD-S2	CTTGAATTCGAGCTCGGTACCGTTGATACCTTGTGGAGTGGG	ATACAGAGCACATGCCTCGAGCGGCTTGCATTGAGCGTGAG	
CsPOD-P1	AATTCGAGCTCGGTACCCGGGTGTGGTTGTGTATTCACGTCCTG	AGCACATGCCTCGAGGTCGACTTGTGCACATAGACCGCAC	
CsPOD-P2	AATTCGAGCTCGGTACCCGGGCCTTCTAGAACTTAAGCGTG	AGCACATGCCTCGAGGTCGACGTTAGATGCGAGATTGAGCTACTC	
CsPOD-P3	AATTCGAGCTCGGTACCCGGGGGATTTCGAATATGTGGAATC	AGCACATGCCTCGAGGTCGACCCCTTAATTAGGTTTATGTG	
CsCAT-C1	CTTGAATTCGAGCTCGGTACCTCCTACAGCCCAGGTCTTCAG	ATACAGAGCACATGCCTCGAGGAATTAAATAACTTTGCGTC	
CsCAT-C2	CTTGAATTCGAGCTCGGTACCCTAGGTATGTACTAAATAGCC	ATACAGAGCACATGCCTCGAGGTGATGATACAGCTACAAACTC	
CsCAT-C3	CTTGAATTCGAGCTCGGTACCCGCTATAGATAGAAAAACGGGTG	ATACAGAGCACATGCCTCGAGCGAGAAGCTACGCTACGAG	

CsbHLH18	CGGGATCCATGTTTCCGTTTCAAAGTCACC	CGGAATTCCTAATCACGATGATCACATTCG	Transient expression assay
CsPOD-P1-0800	CTATAGGGCGAATTGGGTACCGTGGTTGTGTATTCACGTCCTG	CTGCAGGAATTCGATCCCGGGTTGTGCACATAGACCGCAC	
mP1-E1	ATGTATTTTGGCCGCAGTCACTTA	TAAGTGACTGCGGCCAAAATACAT	
mP1-E2	TTTGTCTGATGCCCATAGTTTGAC	GTCAAACTATGGGCATCAGACAAA	
Actin	ATCCCTCAGCACCTTCC	CCAACCTTAGCACTTCTCC	Analysis of expression level
Ubiquitin	GGTGTTTCCAGTGGCGGACG	TCCTCCCCTCAGCTACGGGGTAT	
CsbHLH1	TTCCCAGATGCACAACCTCC	CGGGGGCAAGTTGAGATGAA	
CsbHLH2	GGCCTGGTTCAACAAAGAGG	AGCTTGGTCGCTTGTCCAAT	
CsbHLH3	ATGGCGGAAAGCAATGCAAA	ATTGAACGCAGATGGCAACC	
CsbHLH4	TCAGTGCTGGCGAAGACAAT	CTCACCAGCCTCCCTTTCAC	
CsbHLH5	GAGAGAGATCGACACCCGGA	GCTCAGCTGCCTCTGAACAT	
CsbHLH6	TCAGCTGGTGGTATCGGGTA	TGGTAAGACGCTGGTGTTGG	
CsbHLH7	GTCTCCTGCACCTACAGTCG	ACTAACCCTCCTGGGCTCTT	
CsbHLH8	AACCGACGCTGATGAAGTGA	CCGGCCTCCCAATGTTGATA	
CsbHLH9	ATTCGCTTGCTCCACGAAGA	TGCTGAAGCATCATGGTCCG	
CsbHLH10	GGACCAAAGCCACCCTGTAA	TACCCATTGACCGGGTGTTG	
CsbHLH11	TCGCTTCAGCAACTCACCTC	GAGTGAGATGAGGGCATCGG	
CsbHLH12	GGCTTCTGGAAGTGGGTTGT	TGACTGTCAGTGGCTTGACC	
CsbHLH13	AGAAACGGGTCATCTTCGCA	TCTTCGGTTCCTCTCAGCCT	
CsbHLH14	CGGCACTGAAGCTAGGCTTAT	CATCCTGGTGGAGAATGCGA	

CsbHLH15	TGAAGCTCGTTTCTGGGCTC	TGCAATGTCATCCCCTGAAGT	
CsbHLH16	AGTGCGTCCAAAAGGCAATG	TCAGTTGCCGACCCTCTACT	
CsbHLH17	ACGATCAGTTGTCAGCTCCG	CAGGCAATCAGTCCCCATGT	
CsbHLH18	TGAGAAGCCAAGCTACGGAC	ACCAACCGTCGTCATCTCAG	
CsbHLH19	ATTGGGACTCATCATCATCT	ATTCATTCATCTGCTTACGG	
CsbHLH20	GGTAGCAGTAGCTCAAGCGG	CAAGCTCAGCCCAGCAGTAA	
CsbHLH21	TGGCATCGGGTTGCCTTTAT	GGCATCGATCCTCCCTGAAG	
CsbHLH22	CGGTTCTTTGATTCGGCAGG	AGCGGTGATATTTGCGGTGA	
CsbHLH23	ATCTCTATCGCCGCAAGCTG	TAGAGCAATGGGCATGAGGC	
CsbHLH24	CACTGAATCGCAACGGCAAT	GATTGTATCGGTCGGCCTCC	
CsbHLH25	GAACTACGTGCAAGGGTGGT	AGCAGGCGCTTGAGATAGTG	
CsbHLH26	GCTTCGCAATAACCGCTCTG	ATTGCAGCTTGTGCAGTGTC	
CsbHLH27	GTCATGGGTTCGGGGAAAGT	GCTGCAGAACTTGCTTCCAC	
CsbHLH28	GGTAATGGGTCCCCCGTTAAA	TCGATGGTTTTTCGGTCAGGT	
CsbHLH29	TGACCGAACTGATCCTCACG	GTTTGTTGTTTCTGGGCGCT	
CsbHLH30	GCCTTTCTCCGGGCTAATCT	TCTTGAGCTTGCAAAGGGCT	
CsbHLH31	AACTCATCCAACGCTCCAGG	GAGGCCACCACTAGATCTGC	
CsbHLH32	GACGAGCAGTAGCCACTTGA	TGGCCTTGGCCTACCATTTT	
CsbHLH33	GCACCAGAGGCTATGTCAGG	ACTGCACCATCCTTTGGGTC	
CsbHLH34	GCCCATGATGAGGTGGTTGT	GTCAACTGCTCCGATCCCTG	
CsbHLH35	AATCAGCGACCGGTTCAAGA	GAAGCTGACCGAAAGCAACC	
CsbHLH36	GATTACTCCAGCGACGGGAC	CGTTGCGGATTCTTCCTCCA	
CsbHLH37	GGACTGTCATGGGGATTGGT	TCAACCTTGGAGCCATTGGG	
CsbHLH38	CTGGAATGTTCCCGCAAAGC	CCGGCAAACACTTCTTGTGG	
CsbHLH39	GCGATCCTTGGGATGATGCT	AAATGGTGGACCAAGCGAGT	
CsbHLH40	TCACCAATCCTACCGTGGTC	TGGTTGTGCTGGGGAGTAAG	
CsbHLH41	TGCAGTGTCTTCAACTGAGCA	CAGTTGCTTGACCCCTCCTT	

CsbHLH42	TCAGCCTTTTTCAGCCACCA	TGACGTTTCGCTGTTCGGAT	
CsbHLH43	AGCACTTTTGGGAAGTGCGA	TCTGTTTCTGCCCGTGCTTC	
CsbHLH44	CTGCCAGAAATCGAAGCAAGG	CAACTGGAAAGCTGAGTGCAG	
CsbHLH45	AACAAGCCCGAACTTGGCT	TCGACTCATCTTCCGAGCAG	
CsbHLH46	TCAGGGCAATGGTTCCTTGG	TACCACTTGGCTGTGCAACT	
CsbHLH47	GCTTGAAGGATTTGGGCTCG	GCCTTCCTGGTAGTGACGAA	
CsbHLH48	CTTGGCCCGAACAAAATGGC	CATGAATCACTACGCGCCCT	
CsbHLH49	CTCCTTCACAGCACTCCTCG	AAGTTTGCCCCTGGGTTGAA	
CsbHLH50	GTCACGGGTCTCTGAAGTCG	AGCAGCCATCTCTGCTGAAG	
CsbHLH51	ACAAGCAAATGGCCAGCAAG	ACCAGTAAAAGCCATGCCGA	
CsbHLH52	AGGCAACGGAGGCAAGAAAT	CCCGCAGCTATAGGCAATCT	
CsbHLH53	GCAATGCCGGGTTCAAAGAA	TTTGGCTCGGATTTTGCACG	
CsbHLH54	ACTTGCACTCACCGGTCAAA	GGTGATGGGAGGGGATTTGG	
CsbHLH55	TCCAATGCCCCTACCACTTG	GCAGCCCTTTCAGCAAATCC	
CsbHLH56	CCAGCAGCAAGTCGAGAGAT	ACCTCACAGTCCAGTCCGTA	
NtPOD	CAAATGTAAGAGGAAACTCAGAGG	AGCAACAACTCCAGCTAATTGATAG	
NtSOD	AGCTACATGACGCCATTTCC	CCCTGTAAAGCAGCACCTTC	
NtCAT	AGGTACCGCTCATTCACACC	AAGCAAGCTTTTGACCCAGA	
CsbHLH18-RNAi	CATGATCGTGACTCACCCCAC	CTTTACCGCAGAGCTGACAGT	
CsPOD	TGCTGGACAGCACTAACACC	CCAATGGAACTGCCCATGAC	
CsSOD	CAAGCCGCAATAGCAGCCAT	CCGTGGAATGCAGAGTGAAG	
CsCAT	CTGCCAGTTCTTTCAACGCC	AAACCCTTAGCACTGGCTCC	

Supplementary Table S2. The list of CsbHLH members identified in sweet orange genome.

Number	Name	Gene ID	CDS length (bp)	AA	MW (kDa)	pI	Chromosome position
1	CsbHLH1	orange1.1g009555m	1599	532	60.49	5.91	1
2	CsbHLH2	orange1.1g020172m	993	330	36.59	7.23	1
3	CsbHLH3	orange1.1g043618m	933	310	34.64	6.60	1
4	CsbHLH4	orange1.1g040546m	678	225	23.65	9.93	1
5	CsbHLH5	orange1.1g038389m	1062	353	39.5	5.1	1
6	CsbHLH6	orange1.1g037441m	1101	366	40.71	8.41	1
7	CsbHLH7	orange1.1g026436m	717	238	26.64	8.67	1
8	CsbHLH8	orange1.1g024670m	795	264	28.8	7.65	2
9	CsbHLH9	orange1.1g029765m	567	188	21.04	6.09	2
10	CsbHLH10	orange1.1g011373m	1464	487	52.83	6.01	2
11	CsbHLH11	orange1.1g041370m	933	310	35.15	5.38	2
12	CsbHLH12	orange1.1g042908m	729	242	26.89	6.39	2
13	CsbHLH13	orange1.1g024326m	810	269	30.02	5.84	2
14	CsbHLH14	orange1.1g027547m	669	222	25.15	5.83	2
15	CsbHLH15	orange1.1g036531m	954	371	35.82	5.19	3
16	CsbHLH16	orange1.1g036928m	1101	366	40.67	5.2	3
17	CsbHLH17	orange1.1g018897m	1050	349	39.49	4.7	3
18	CsbHLH18	orange1.1g024930m	783	260	28.92	7.09	3
19	CsbHLH19	orange1.1g020210m	990	329	36.41	5.72	3
20	CsbHLH20	orange1.1g023215m	858	285	31.29	8.29	3
21	CsbHLH21	orange1.1g012156m	1413	470	49.24	5.83	4
22	CsbHLH22	orange1.1g025665m	750	249	28.96	8.84	4
23	CsbHLH23	orange1.1g010045m	1560	519	56.32	4.96	4

24	CsbHLH24	orange1.1g008967m	1644	547	61.07	6.36	4
25	CsbHLH25	orange1.1g026212m	726	241	26.54	5.33	5
26	CsbHLH26	orange1.1g048534m	276	91	10.32	9.09	5
27	CsbHLH27	orange1.1g023847m	831	276	29.82	5.51	5
28	CsbHLH28	orange1.1g037136m	513	170	19.27	9.46	5
29	CsbHLH29	orange1.1g047562m	741	246	28.26	6.32	5
30	CsbHLH30	orange1.1g038298m	780	259	29.23	9.35	5
31	CsbHLH31	orange1.1g038558m	1110	369	39.14	8.65	5
32	CsbHLH32	orange1.1g007258m	1836	611	67.22	5.97	5
33	CsbHLH33	orange1.1g010728m	1509	502	55.64	6.16	5
34	CsbHLH34	orange1.1g048817m	1842	613	68.14	5.95	5
35	CsbHLH35	orange1.1g039972m	510	169	18.99	7.11	6
36	CsbHLH36	orange1.1g026479m	717	238	26.94	7.01	6
37	CsbHLH37	orange1.1g038918m	876	291	32.23	6.33	6
38	CsbHLH38	orange1.1g008631m	1677	558	59.55	5.9	7
39	CsbHLH39	orange1.1g016638m	1158	385	42.37	7.06	7
40	CsbHLH40	orange1.1g037785m	1098	365	40.77	6.46	7
41	CsbHLH41	orange1.1g017497m	1113	370	41.6	6.01	7
42	CsbHLH42	orange1.1g044652m	1662	553	60.02	6.25	7
43	CsbHLH43	orange1.1g023629m	840	279	30.99	8.85	7
44	CsbHLH44	orange1.1g021208m	951	316	35.28	7.76	8
45	CsbHLH45	orange1.1g047654m	1140	379	41.48	4.93	8
46	CsbHLH46	orange1.1g022288m	900	299	32.99	6.17	8
47	CsbHLH47	orange1.1g026746m	705	234	26.62	6.4	9
48	CsbHLH48	orange1.1g028060m	645	214	24.46	5.52	9
49	CsbHLH49	orange1.1g015330m	1230	409	45.17	6.41	9
50	CsbHLH50	orange1.1g018007m	1089	362	39.89	9.08	9

51	CsbHLH51	orange1.1g043851m	1164	387	43.75	5.96	9
52	CsbHLH52	orange1.1g036406m	723	241	27.33	5.83	9
53	CsbHLH53	orange1.1g015337m	1227	408	45.95	6.9	Un
54	CsbHLH54	orange1.1g014958m	1248	415	47.09	5.61	Un
55	CsbHLH55	orange1.1g011621m	1446	481	52.72	6.67	Un
56	CsbHLH56	orange1.1g025477m	759	252	28.84	7.64	Un

Supplementary Figures

Figure S1. Gene structures of the *CsbHLH* genes.

Figure S2. Expression patterns of CsbHLH genes in response to cold stress, as analyzed by RT-PCR.

Figure S3. Generation and molecular identification of transgenic tobacco plants overexpressing *CsbHLH18*.

Figure S4. Alignment of nucleotide sequences of bHLH18 from sweet orange and trifoliate orange.

Figure S5. Transformation and molecular characterization of trifoliate orange expressing an RNAi vector.

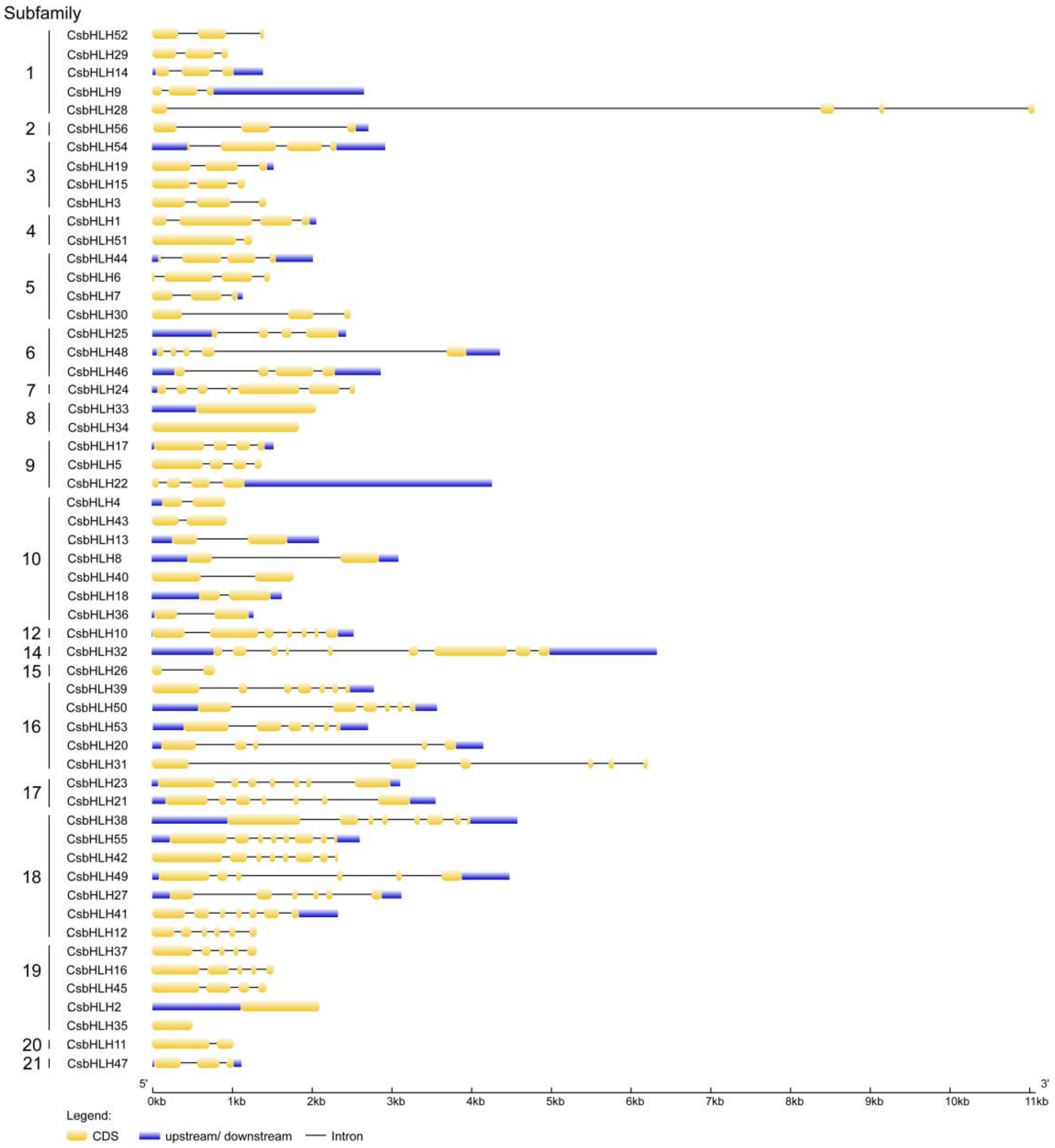

Figure S1. Gene structures of the *CsbHLH* genes. Subfamily numbers were listed on the left. The exons, introns and untranslated regions were replaced by the yellow rectangles, black lines and blue rectangles respectively.

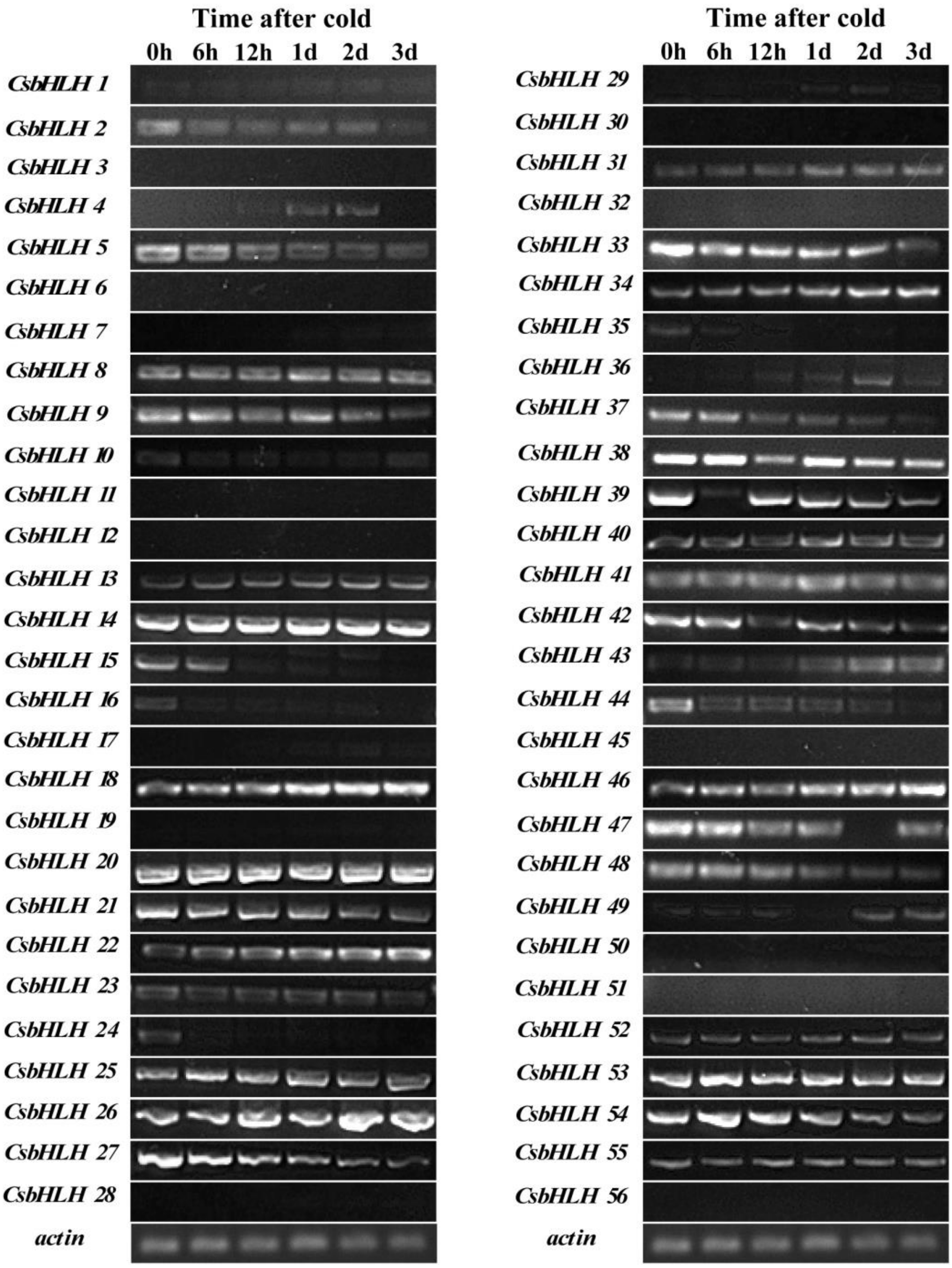

Figure S2. Expression patterns of *CsbHLH* genes in response to cold stress, as analyzed by RT-PCR. *Actin* was used as an internal control.

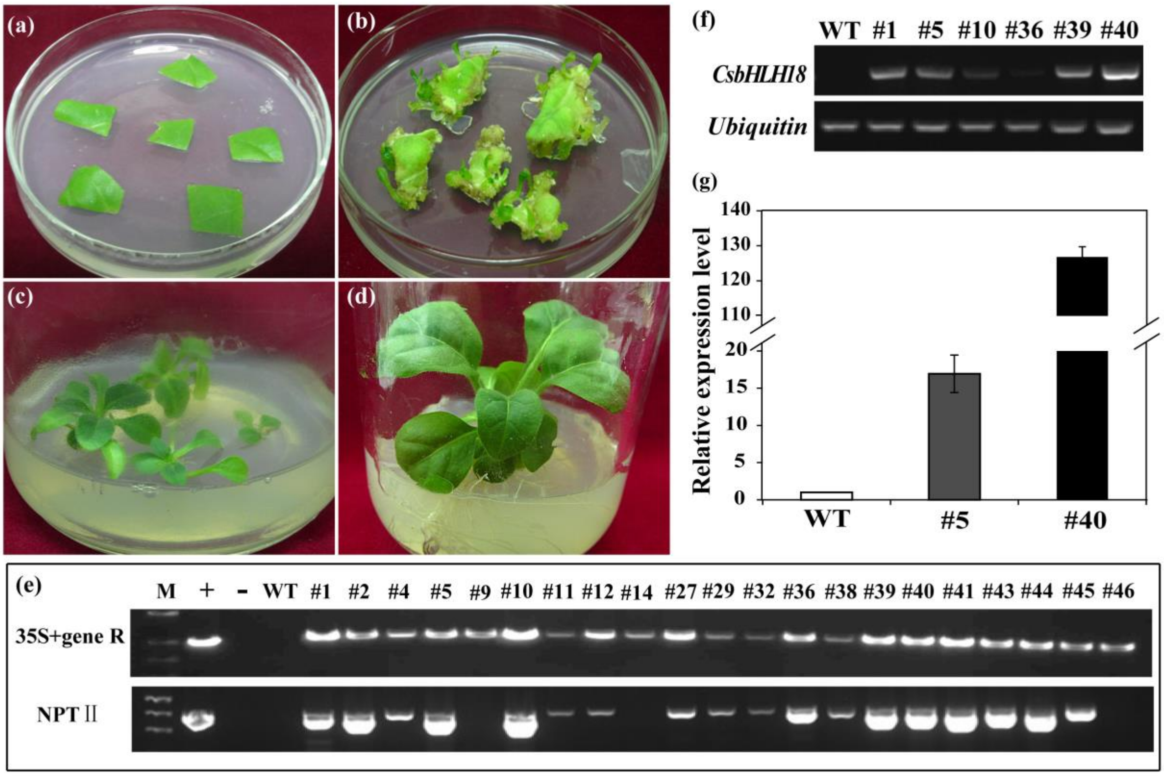

Figure S3. Generation and molecular identification of transgenic tobacco plants overexpressing *CsbHLH18*. (a). Co-culture of tobacco leaf discs; (b). Growth status on the selected medium for one month; (c). Regeneration buds; (d). Rooted plant. (e). PCR identification of the regenerated buds using *CaMV35S-CsbHLH18* primers and *NPTII* (neomycin phosphotransferase II) primers one the upper panel and lower panel respectively. M: DNA Marker; +: plasmid; -: H_2O; WT: wild type; the numbers on the top indicated different transgenic lines. (f-g): Expression analysis of *CsbHLH18* in transgenic lines by semi-qPCR and qRT-PCR respectively. *Ubiquitin* was used as an internal control.

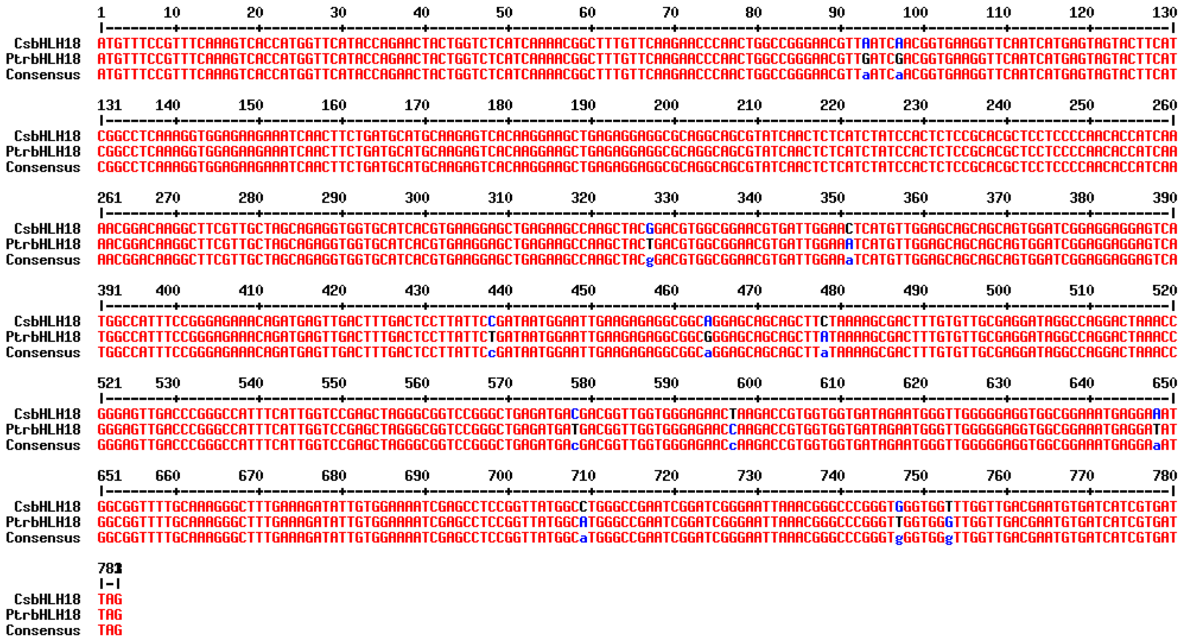

Figure S4. Alignment of nucleotide sequences of bHLH18 from sweet orange and trifoliate orange.

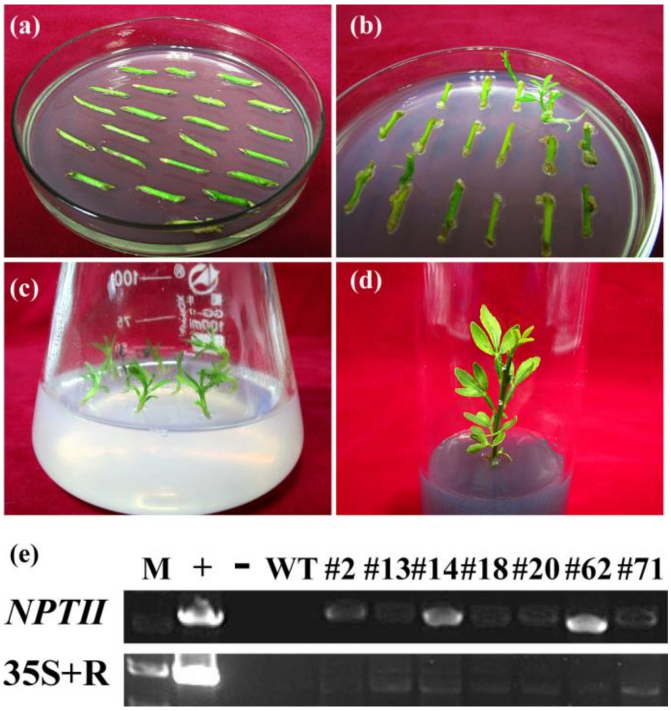

Figure S5. Transformation and molecular characterization of trifoliate orange expressing an RNAi vector. (a-d) Regeneration processes of trifoliate orange shoot stems after *Agrobacterium*-mediated transformation of the RNAi vector. (e) Genomic PCR identification of the plants using specific primers of *NPTII* (upper panel) and *35S+bHLH18* (bottom panel). M: DNA Marker; +: plasmid; -: H_2O; WT: wild type. Numbers on the top of the two gel panels indicate the transgenic lines used for analysis.
